# Factors Affecting the Running Performance of Soccer Teams in the Turkish Super League

**DOI:** 10.3390/sports12070196

**Published:** 2024-07-19

**Authors:** Spyridon Plakias, Yiannis Michailidis

**Affiliations:** 1 Department of Physical Education and Sport Science, University of Thessaly, 38221 Trikala, Greece; spyros_plakias@yahoo.gr; 2 Laboratory of Evaluation of Human Biological Performance, New Buildings of Laboratories, Department of Physical Education and Sports Sciences, Aristotle University of Thessaloniki, 57001 Thessaloniki, Greece

**Keywords:** football, performance analysis, tactics, formations, running performance metrics, contextual variables

## Abstract

Performance analysis in sports is a rapidly evolving field, where academics and applied performance analysts work together to improve coaches’ decision making through the use of performance indicators (PIs). This study aimed to provide a comprehensive analysis of factors affecting running performance (RP) in soccer teams, focusing on low (LI), medium (MI), and high-speed distances (HI) and the number of high-speed runs (NHI). Data were collected from 185 matches in the Turkish first division’s 2021–2022 season using InStat Fitness’s optical tracking technology. Four linear mixed-model analyses were conducted on the RP metrics with fixed factors, including location, team quality, opponent quality, ball possession, high-press, counterattacks, number of central defenders, and number of central forwards. The findings indicate that high-press and opponent team quality affect MI (d = 0.311, d = 0.214) and HI (d = 0.303, d = 0.207); team quality influences MI (d = 0.632); location and counterattacks impact HI (d = 0.228, d = 0.450); high-press and the number of central defenders affects NHI (d = 0.404, d = 0.319); and ball possession affects LI (d = 0.287). The number of central forwards did not influence any RP metrics. This study provides valuable insights into the factors influencing RP in soccer, highlighting the complex interactions between formations and physical, technical–tactical, and contextual variables. Understanding these dynamics can help coaches and analysts optimize team performance and strategic decision making.

## 1. Introduction

Performance analysis in sports is a great and rapidly evolving field [1]. On one hand, academics continually provide new research data, while on the other, applied performance analysts (PAs) join the coaching staff of teams, assisting in improving coaches’ decision making [2,3,4]. PAs use performance indicators (PIs) to draw conclusions [5]. PIs can be categorized based on whether their data originate from event or positional-tracking data [6], while to strengthen the meaningfulness of these, the importance of contextual variables has been highlighted [7]. Event data provide information on technical and tactical issues [8], tracking data are useful for analyzing running performance (RP) and tactics [9], while contextual variables enhance the quality of both [10]. Running performance (RP) is a crucial element of the overall performance of soccer players, contributing along with technical, tactical, and mental components [11]. However, on its own, it is not sufficient to interpret match outcomes [12,13], and it can be influenced by many factors [14]. Therefore, when interpreting RP in soccer, one should include contextual variables that may also explain part of the mental components [15,16], as well as technical–tactical variables. Such a holistic approach can provide genuinely useful information to team coaches.

The role of contextual variables in the RP of teams has been documented by research for many years, as Lago-Peñas [17] states in his review article. Regarding the most recent research, several contradictory findings have emerged. Modric et al. [18] found that away matches were associated with increased total distance (TD) and low-intensity (<4 m/s) running. Jerkovic et al. [19] found a greater amount of distance covered in the running zone (4–5.48 m/s) for away matches, while Gonçalves et al. [20] observed higher values of high-intensity running (60–100% of the individual maximum running speed) in home matches compared to away matches. Additionally, Modric et al. [18] found that team quality was not associated with RP, whereas Aquino et al. [21] reported that the top-ranked team covered greater total distance with high acceleration compared to the bottom-ranked team. Finally, regarding opponent quality, Modric et al. [18] and Jerkovic et al. [19] concluded that the quality of the opponent team was not associated with RP. Conversely, Gonçalves et al. [22] found that starters playing against strong opponents exhibited higher values of distance in high-speed running (5.5–7 m/s), and Gonçalves et al. [20] found that matches against strong opponents resulted in greater total distance covered.

However, analyzing situational variables in isolation seems to offer only a limited understanding of the complicated nature of team sports performance, since research has shown that ball possession, as well as other technical and tactical actions, is also affected by the location of the match and the opponents’ strength [17]. In examining the effects of tactical behavior, several researchers have studied the impact of ball possession on RP. For example, Modric et al. [23] found that in the UEFA Champions League 2020–2021, teams with high ball possession covered more distance in high-speed running (5.5–7 m/s) and sprinting (>7 m/s) than teams with low ball possession. In contrast, da Mota et al. [24] found that in the 2014 FIFA World Cup, teams with high ball possession covered similar distances at medium (3–3.9 m/s) and high speeds (>3.9 m/s) but covered greater distances in total and at low speed (≤3 m/s) compared to teams with low ball possession. Additionally, Modric et al. [23] found no differences in counterattacks and high-pressing regardless of whether teams covered greater or smaller distances both overall and at high intensities. Conversely, Forcher et al. [25] identified differences in RP between teams that adopt a counterattacking style compared to those that favor a ball possession style, and Low et al. [26] found differences in RP between teams employing deep-defending and high-press strategies. These studies highlight the complex and varied effects of tactical behaviors on RP in soccer, indicating that different styles of play can significantly influence physical demands on players, something that also emerged in the research of Plakias et al. [27], who made a direct comparison between two opposite styles in 19 tactical situations.

Finally, regarding formations, which constitute another tactical aspect of soccer [28], Modric et al. [29] demonstrated that the values for almost all of the RP metrics are greater with three central defenders than with two central defenders. This conclusion was also reached by Tierney et al. [30], who found greater distances covered at high speeds (≥5.5 m/s) with three central defenders. Additionally, Borghi et al. [31] found that formations with two central forwards cover greater distances at high intensities (>5.5 m/s) than those with one central forward (3-5-2 > 4-4-2 > 4-3-3). This finding aligns with the results of Bradley et al. [32] (4-4-2 > 4-3-3 > 4-5-1) and Arjol-Serrano et al. [33] (4-4-2 > 4-2-3-1), but not with those of Aquino et al. [34] and Vieira et al. [35], who found that mean speed and high-intensity activities are greater in the 4-3-3 formation compared to the 4-4-2. It is worth noting that all the aforementioned studies on formations had sample sizes ranging from 20 to 59 matches, limiting the generalizability of the conclusions regarding formations. For this reason, the authors focused more on the differences between player positions.

All the aforementioned conflicting findings indicate that RP may be influenced differently in each competition, depending on the level and type of competition. Additionally, examining factors in isolation may yield some results, but it cannot explain the complex nature of soccer, where physical, technical–tactical, and contextual factors interact with each other. Building on the existing literature, we hypothesized that a combination of eight factors, including location, team quality, opponent team quality, ball possession, high-press, counterattacks, number of central defenders, and number of central forwards, significantly affect various aspects of running performance. Specifically, we anticipate that these factors will differentially impact low-speed distance (LI), medium-speed distance (MI), high-speed distance (HI), and the number of high-speed runs (NHI).

Using a large sample of matches that included all teams from the first Turkish league in the 2021–2022 season, we aimed to address an additional gap in the existing literature. Specifically, most studies examining factors affecting team RP often rely on samples from a single team with GPS data access or a limited number of matches from international competitions like the UEFA Champions League or the FIFA World Cup. Therefore, the purpose of this study is to provide a comprehensive analysis of the factors (including formations, technical–tactical variables, and contextual factors) that affect the RP of soccer teams, focusing on LI, MI, HI, and NHI. This holistic approach aims to enhance the understanding of how different elements interact to affect RP in soccer. By doing so, it seeks to fill the existing gaps in the literature and offer practical insights for optimizing team performance at a competitive level. Understanding these dynamics can help coaches and analysts design better training and game strategies, ultimately improving team performance and success in matches.

## 2. Material and Methods

### 2.1. Sample

This study analyzed data from the Turkish first division’s 2021–2022 season, encompassing 20 teams over 38 matchdays, with each matchday featuring 10 games. Instatscout supplied data for the first 24 matchdays, covering 240 matches. Due to missing data for two matches and the exclusion of 53 matches because of red card incidents, the final sample included 185 matches, resulting in 370 (i.e., 2 × 185) observations, with each team in a match providing one observation.

### 2.2. Procedure

Data collection of the running variables was performed using InStat Fitness’s optical tracking technology (https://football.instatscout.com/, accessed on 1 May 2024), a FIFA-certified system known for its high precision and reliability, as confirmed on the FIFA website [36] and referenced in previous studies [37,38]. For the 2021–2022 season, InStat’s system was designated as the official Electronic Performance and Tracking System (EPTS) for the league [27]. For the technical–tactical variables the data were obtained from the Instatscout platform. As reported in previous studies, the reliability of Instatscout data is very high (K values 0.90 to 0.98) [39,40,41].

### 2.3. Ethics

Ethics committee approval of the current study was gained from the University of Thessaly (No. 1973, 12 October 2022). Additionally, InStat Ltd. granted written consent on 8 November 2022, for the utilization of the data in this research, ensuring adherence to all ethical standards for research and publication.

### 2.4. Statistical Analyses

Initially, cluster analysis was applied to create categorical variables concerning: (a) the quality of the teams and the opponents as strong or weak (based on the number of points each team collected in the final championship standings), (b) ball possession as high or low (based on the team’s possession percentage in each observation), (c) the counterattacks of the match as many or few (based on the total number of counterattacks for both teams in the match for each observation), and (d) the high-pressing actions as many or few (based on the number of high-pressing actions of the team in each observation).

Next, after checking the normality of the distribution of the four dependent variables shown in Table 1, a linear mixed-model analysis was performed four times, correspondingly. In all cases, the eight independent variables shown in Table 1 were used as fixed factors, and the variable TEAM was used as a random factor. The definitions in Table 1 for high press, counterattacks, and ball possession are derived from the glossary of Instatscout, the platform from which the data for the respective variables were obtained. The variable TEAM represented the 20 different teams participating in the Turkish league in the 2021–2022 season. The thresholds for creating the running variables are those used in previous research [27,29,42]. However, in this study, the intensities of standing (<0.2 m/s), walking (0.21–2 m/s), and jogging (2.01–4 m/s) were combined as low-intensity, the intensity of running (4.01–5.5 m/s) was named medium-intensity, and the intensities of high speed (5.51–7 m/s) and sprint (>7 m/s) were combined as high-intensity. All statistical analyses were conducted using the IBM SPSS statistical package (version 29.00, IBM Corporation, Armonk, NY, USA), with a significance level set at *p* < 0.05. Cohen’s d was utilized to measure the effect size. The effect sizes were defined as follows: trivial (d = 0.0 to 0.19), small (d = 0.2 to 0.49), medium (d = 0.5 to 0.79), and large (d ≥ 0.8) [43].

## 3. Results

### 3.1. Cluster Analyses

Based on the number of points they accumulated in the championship, the teams were classified into two categories (strong/weak). The strong category included teams that ranked 1st–13th with points ranging from 52 to 81 (cluster center 61.00), while the weak category included teams that ranked 14th–20th with points ranging from 20 to 47 (cluster center 36.57).

Based on the number of high-pressing actions performed by the teams in each match, the observations were classified into two categories (many/few). The many category included observations where the team had 10 to 21 high-pressing actions (cluster center 12.50), while the few category included observations where the team had 0 to 9 high-pressing actions (cluster center 5.96).

Based on ball possession percentage, the observations were classified into two categories (high/low). The high category included observations where the team had 50.01% to 80% possession (cluster center 56.54), while the low category included observations where the team had 20% to 49.99% possession (cluster center 42.67).

Finally, based on the number of counterattacks performed by both teams combined in each match, the observations were classified into two categories (many/few). The many category included observations where the match had 30 to 53 counterattacks (cluster center 34.76), while the few category included observations where the match had 13 to 29 counterattacks (cluster center 23.74).

### 3.2. Linear Mixed Models

Table 2 shows the means of the dependent variables across the categories of the independent variables. Table 3 shows F and p values of the type III tests of fixed effects for the four models that were generated for the corresponding four dependent variables.

From Table 2 and Table 3 it appears that:

- LI is greater for the high BP group compared to the low BP group (*p* = 0.009, d = 0.287).
- MI is greater for the many HP group compared to the few HP group (*p* = 0.002, d = 0.311).
- MI is greater for teams when facing strong opponents compared to when facing weak opponents (*p* = 0.024, d = 0.214).
- MI is greater for strong group compared to weak (*p* = 0.04, d = 0.632).
- HI is greater for home matches compared to away matches (*p* = 0.014, d = 0.228).
- HI is greater for teams in matches with many CA compared to matches with few CA (*p* < 0.001, d = 0.450).
- HI is greater for the many HP group compared to the few HP group (*p* = 0.004, d = 0.303).
- HI is greater for teams when facing strong opponents compared to when facing weak opponents (*p* = 0.041, d = 0.207).
- NHI is greater for the many HP group compared to the few HP group (*p* < 0.001, d = 0.404).
- NHI is greater for teams playing with three central defenders compared to teams playing with two central defenders (*p* = 0.038, d = 0.319).

## 4. Discussion

The purpose of this study was to investigate the factors influencing the distances covered by soccer players at three different intensity levels, as well as the number of high-intensity runs. Our findings indicate that high-press and opponent team quality affect MI, HI, and NHI; team quality affects MI; location and counterattacks influence HI; the number of central defenders affects NHI; and ball possession impacts LI. Conversely, the number of central forwards does not appear to influence any of the RP metrics. These findings can inform tactical decision making by coaches, taking into account the physical condition of their team and the context of the match.

### 4.1. Contextual Variables

In this study, we found that location has a statistically significant effect on high-intensity running, with home matches displaying higher HI values. This finding aligns with Gonçalves et al. [20], who observed higher values of high-intensity running in home matches compared to away matches. It appears that the psychological boost from the home crowd motivates players to exert more effort, engaging in more high-intensity actions [15,44]. Other studies have found that in the UEFA Champions League, away matches were associated with increased low-intensity running [18], and in the Croatian first division, higher medium-intensity values were found for away matches [19], differences that do not seem to apply to the Turkish league.

Additionally, our research found that strong teams exhibit higher MI values, which contradicts the findings of Modric et al. [18], who found that team quality was not associated with running performance in the UEFA Champions League. However, a year earlier, another study by Aquino et al. [21] found that the top-ranked teams covered greater total distance and higher acceleration than the bottom-ranked teams in a national league setting (Brazilian 2nd Division League).

Finally, our study found that facing a strong opponent increases the values of MI, HI, and NHI, compared to facing a weak opponent. This finding is consistent with the research by Gonçalves et al. [20] and Gonçalves et al. [22] for the first division of the Brazilian championship and the U20 Brazilian National League, respectively. The former study showed that matches against strong opponents resulted in greater total distance covered, while the latter found that starters playing against strong opponents exhibited higher values of distance in high-speed running. It seems that facing a strong opponent is a factor that increases intrinsic motivation [45].

### 4.2. Technical–Tactical Variables

In our study, we found that teams with high ball possession percentages exhibit higher LI compared to teams with low ball possession percentages, without statistically significant differences in other RP variables. This finding aligns with da Mota et al. [24], who found that teams with high ball possession covered greater total distances and distances at low intensities compared to teams with low ball possession. Additionally, we discovered that teams involved in matches with many counterattacks (regardless of whether they are performed by themselves or their opponents) have higher values in HI. The results of Külah and Alemdar [46], where the authors found that teams focused on ball possession had a lower average sprint value than teams playing a counterattacking style, agree with our findings. Counterattacks are, indeed, high-intensity actions [47], and high-speed training can be performed within the specific context of counterattacks [48].

Finally, we found that teams engaging in many high-pressing actions exhibit higher values in MI, HI, and NHI compared to teams with fewer high-pressing actions. These findings align with the assertion of Morgans et al. [49] that a defensive tactical strategy that does not promote a “high-press” but maintains a compact defensive shape reduces high metabolic load distance (HMLD), which provides overall information about the soccer players’ high-intensity activities (>25.5 W/kg) [50,51,52]. Additionally, Low et al. [53] showed that using a high-press defending strategy leads to larger dispersion, due to a longer team length and larger interline distances between defenders, midfielders, and forwards. Consequently, covering these larger distances requires greater physical effort. This is further supported by Abreu et al. [54], who found that high-press games in soccer training elicited higher physiological and physical responses than free play.

### 4.3. Formations

In our study, we found that NHI increases when teams play with three central defenders compared to when they play with two. This finding is consistent with those of Tierney et al. [30] and Modric et al. [29], who also reported greater distances covered at high speeds for teams using three central defenders. These results align with the conclusions drawn from the systematic review of Forcher et al. [55], who found that physical match performance was higher in formations with three defenders (e.g., 3-5-2) compared to formations with four defenders (e.g., 4-4-2).

Additionally, our research did not find any effect of the number of central forwards on RP metrics. This specific aspect has not been directly investigated in previous studies. Indirect conclusions from research that studied overall formations are contradictory; some studies suggest that formations with two central forwards cover greater distances at high intensities [31,32,33], while others indicate that mean speed and high-intensity activities are greater in the 4-3-3 formation compared to the 4-4-2 formation [34,35].

### 4.4. Limitations

This study has some limitations that should be acknowledged. Firstly, the sample is exclusively drawn from the Turkish first division during the 2021–2022 season. This geographical and competitive context limits the generalizability of our findings to other leagues or levels of play, where different styles of play [56,57,58] may influence running performance differently. Secondly, the data collection method used in this study was static, meaning that data were obtained after the completion of matches [59]. This contrasts with a dynamic method, which would involve real-time data acquisition and could account for changes in match status (e.g., goals scored, red cards, substitutions) as they occur [60]. Moreover, while this study included several important factors influencing RP, there are other potential variables (such as weather conditions, playing surface, seasonal variations, mid-season coach change, and fatigue) that were not considered and could also impact running metrics [61,62,63,64,65,66]. However, to the best of our knowledge, no other study has investigated the effect of so many factors, including formations, technical–tactical, and contextual variables, by simultaneously studying them in multivariate models and using a large sample of 185 high-level matches. In addition, the static method, despite its drawbacks, has been widely used in performance analysis and has provided useful information [37,59].

## 5. Conclusions

In summary, our study aimed to investigate the factors influencing RP in Turkish soccer teams, focusing on different intensity levels and the number of high-intensity runs. By analyzing a large sample of matches from the Turkish first division during the 2021–2022 season, we identified several key factors that significantly impact RP, including location, team quality, opponent team quality, ball possession, high-press, counterattacks, and the number of central defenders. These findings offer a comprehensive understanding of how various elements interact to affect physical performance in Turkish soccer, providing valuable insights for optimizing team performance.

The primary strengths of our research include the extensive dataset of 185 high-level matches and the multivariate approach that simultaneously examined multiple factors. This holistic methodology allows for a more nuanced analysis of the interactions between different variables influencing RP. However, the study is limited by its focus on a single league, which may restrict the generalizability of the findings. Additionally, the static method of data collection used, while common in performance analysis, does not account for dynamic changes during the match, such as shifts in match status.

The importance of our research lies in its potential impact on both the specialized scientific community and the broader public. For sports scientists and PAs, our findings contribute to the ongoing discourse on the determinants of RP in soccer. For coaches and team managers, understanding these dynamics can inform strategic decisions and training programs, ultimately enhancing team performance and competitive success.

Practically, our results can be applied to improve tactical decision making in soccer. For instance, recognizing that high-pressing actions lead to higher RP metrics can help coaches design training sessions that mimic match conditions and prepare players for the physical demands of competitive play. This can involve specific exercises where players are required to press the opposition in defined zones of the field, followed by rapid counterattacks. Additionally, insights into the effects of opponent quality and match location can guide prematch preparations and in-game adjustments to optimize performance. For example, if a team is playing at home against a strong opponent, the coach might prioritize high-intensity training sessions to boost players’ physical readiness and employ strategies to exploit the psychological advantage of the home crowd. Conversely, for away matches against weaker opponents, the focus might shift towards maintaining possession and managing the game’s tempo to conserve energy and minimize the physical load on players.

Future research should aim to expand on our findings by incorporating data from multiple leagues and varying levels of play to enhance the generalizability of the results. Furthermore, adopting dynamic data collection methods could provide a more accurate depiction of RP by accounting for real-time changes during matches. Investigating additional variables, such as weather conditions, playing surface, and seasonal variations, could also offer a more comprehensive understanding of the factors affecting RP in soccer. Particular emphasis should be placed by the authors on the players’ running under conditions of high temperatures and humidity, as these can have negative consequences, even on the health of the football players [67,68,69].

In conclusion, our study provides a detailed analysis of the factors influencing RP in Turkish soccer, highlighting the complex interplay between physical, technical–tactical, and contextual variables. By addressing the gaps in the existing literature and offering practical applications, we hope to contribute to the advancement of performance analysis in soccer and support the ongoing efforts to optimize team performance and success.

## Figures and Tables

**Table 1:** The variables used when performing the four linear mixed-model analyses.

Type	Abbreviation	Definition
Dependent variables	LI	Low-intensity (0–4 m/s) distance
	MI	Medium-intensity (4.01–5.5 m/s) distance
	HI	High-intensity (>5.51 m/s) distance
	NHI	Number of high-intensity runs (>5.51 m/s)
Independent variables (fixed factors)	LC	Location (home/away)
	TQ	Team’s quality (strong/weak)
	OQ	Opponent’s quality (strong/weak)
	CD	Number of central defenders (two/three)
	CF	Number of central forwards (one/two)
	HP	High-press (many/few): Pressing until 30 m from the opponent’s goal. Pressing, in its turn, is counted for the opponents of a team that is building its attack when players are actively trying to get the ball back.
	BP	Ball possession (high/low): Percentage share of one team’s ball possession in the total ball-in-play time. Ball possession is the sum of all time periods between the start of possession to the moment of transition or to the moment the ball went out.
	CA	Counterattacks (many/few): Attack from the open play that starts with winning the ball from a defensive position and then quickly transitioning to offense while the prior attacking team is caught in an offensive formation; the length of possession during the attack cannot exceed 8 s before the possession transitions or ends; alternatively, the length of possession can last between 8 and 30 s, but the speed of attack cannot be less than 2.6 m/s. A counterattack cannot begin with a pass from a goalkeeper if he controlled the ball for more than 4 s before the action.
Independent variable (random factor)	TEAM	The 20 teams that participated in the Turkish first division 2021–2022 season.

**Table 2:** The mean of the dependent variables across the categories of the independent variables.

Independent Variables	Independent Variables’ Categories	Dependent Variables			
		LI	MI	HI	NHI
**CD**	Two CDs	85,192.247	18,740.194	9780.509	632.877
	Three CDs	85,224.288	19,181.388	10,078.614	652.853
CF	One CF	85,132.495	18,919.433	9969.323	645.492
	Two CFs	85,284.040	19,002.149	9889.800	640.238
TQ	Strong	85,489.889	19,456.661	10,089.752	650.853
	Weak	84,926.646	18,464.921	9769.370	634.877
OQ	Strong	85,239.146	19,129.081	10,043.755	648.218
	Weak	85,177.389	18,792.501	9815.367	637.512
HP	Many	85,117.847	19,205.067	10,096.827	655.501
	Few	85,298.688	18,716.515	9762.295	630.228
BP	High	84,801.273	18,967.825	9991.655	644.705
	Low	85,615.262	18,953.757	9867.467	641.024
CA	Few	85,304.201	18,946.646	9680.661	640.152
	Many	85,112.334	18,974.936	10,178.46	645.578
LC	Home	85,320.289	19,026.764	10,055.527	647.732
	Away	85,096.246	18,894.818	9803.596	637.998

**Table 3:** F and p values of the type III tests of fixed effects for the four models that were generated for the corresponding four dependent variables.

	LI		MI		HI		NHI	
	F	Sig.	F	Sig.	F	Sig.	F	Sig.
Intercept	63,670.426	0.000	6240.045	0.000	4245.507	0.000	5562.454	0.000
CD	0.005	0.943	3.717	0.055	3.108	0.079	4.350	0.038
CF	0.120	0.729	0.146	0.703	0.241	0.624	0.328	0.567
TQ	0.925	0.348	4.878	0.040	1.333	0.262	1.037	0.321
OQ	0.042	0.839	5.129	0.024	4.195	0.041	2.872	0.091
HP	0.338	0.561	10.213	0.002	8.521	0.004	15.153	0.000
BP	6.855	0.009	0.008	0.927	1.160	0.282	0.318	0.573
CA	0.435	0.510	0.039	0.844	21.412	0.000	0.793	0.374
LC	0.652	0.420	0.940	0.333	6.085	0.014	2.830	0.093

## Data Availability

Data are available upon request from the corresponding author due to privacy and ethical restrictions.
